# Feasibility study of ultra-low-head hydro turbines for energy extraction from shallow waterways

**DOI:** 10.1016/j.heliyon.2024.e35008

**Published:** 2024-07-23

**Authors:** Mohamed Murshid Shamsuddeen, Mohammad Abu Shahzer, Min-Su Roh, Jin-Hyuk Kim

**Affiliations:** a Convergence Manufacturing System Engineering (Green Process and Energy System Engineering), University of Science & Technology, Daejeon, South Korea; b Carbon Neutral Technology R&D Department (Fluid Machinery), Korea Institute of Industrial Technology, Cheonan, South Korea

**Keywords:** Ultra-low-head, Hydrokinetic turbine, Screw turbine, Savonius turbine, Gate turbine, Computational fluid dynamics

## Abstract

Ultra-low-head turbines can harness energy from previously deemed unsuitable sites, including natural and man-made locations like shallow estuaries, marine canals, and industrial waterways. Various hydro-turbine concepts were evaluated for their potential to extract power from these areas. These turbines can generate renewable energy for utilization in remotely located areas. A horizontal-axis screw turbine concept, horizontal and vertical Savonius turbine concepts, axial turbine concepts, and a gate turbine concept were investigated in the present study using computational fluid dynamic tools. Reynolds Averaged Navier Stokes equations with a shear stress transport model are used to calculate the flow field. The numerical methodology is then verified with previously published data. The turbine performances were compared and the design feasibility was analyzed to find the most effective turbine design which can extract the maximum energy. The gate turbine concept exhibited a significant power output with high efficiency while the screw turbine showed the lowest performance among the tested designs. The horizontal Savonius turbine displayed enhanced performance with an increment of 23.25 % compared to the screw turbine. An additional parametric study is conducted on the gate turbine namely, the number of runner blades, and the gate installation angle. The 3-bladed gate turbine installed at a 14° gate angle showed superior power output and efficiency than other hydrokinetic turbines.

## Highlights

• Ultra-low-head turbines can obtain power from sites previously deemed unsuitable.
• Several hydro turbine concepts were studied using computational fluid dynamics.
• Screw turbine, Savonius turbine, axial turbine and Gate turbine were investigated.
• The gate turbine concept showed the highest power output with high efficiency.
• Power output from the gate turbine can be controlled by governing the gate angle.

## 1 Introduction

Hydropower (HP) is regarded as a viable renewable energy source in spite of its detrimental effects on the environment. The environmental concern is a key obstacle to the development of bigger and mid-sized HP projects [1]. As a result, there has been a growing interest in small-scale HP resources, which offer benefits such as compact size, simple deployment, and advantageous impact on the environment [2]. Most of the literature that has been made available thus far concentrates on small HP technologies using low hydraulic heads (H) of three to 30 m [3,4], or on hydrokinetic energy transformation technology [5,6]. However, due to the limited economic benefits of the resources, the growth of hydraulic energy in scenarios of 0–1.5 m working H, generally called ultra-low head (ULH) has not received significant attention [7]. Since the energy resources of ULH are geographically dispersed around the globe, it would be challenging to perform a survey for accurate resources [8]. The ULH water energy sites can be identified as; canals, locks, tailrace flow from the tidal/hydropower station, wastewater treatment station canals, streams, marine navigation channels, and seawalls.

Singh and Kasal [9] categorized the HP based on the power output (PO) as medium hydro (15–100 MW), small-hydro (1–15 MW), mini-hydro (100 kW-1 MW), micro-hydro (5 kW–100 kW), and pico-hydro (less than 5 kW). Furthermore, they can be classified based on the H as medium-head (H < 15 m), low-head (H < 5 m), very-low head (H < 2.5 m), and ultra-low-head (H < 1.5 m) [10,11]. When the flow velocity exceeds 0.5 m/s with minimal H or the hydraulic H is less than 1.5 m, the circumstances are referred to be ULH hydraulic energy. Several hydrokinetic turbine technologies and a few HP turbine technologies fall under the ULH category. Through improvements in turbine technologies, streamlined civil construction, and decreased project costs; ULH HP has become a desirable, sustainable, and renewable resource. This form of energy conversion is well adopted since it can be installed close to human activity and is usually thought to be ecologically friendly. Two important factors, in particular, demonstrate the minimal environmental effect of ULH HP: since there is no dam, there are no obstacles to fish migration or navigation, and downstream flow of water is ensured. This is due to the broad blade channels and low rotation speed.

Due to their regulated, predictable, and comparatively clean properties, manmade and natural waterways exhibiting flow rates (Q) greater than 1.5m/s make excellent locations for ULH energy conversion [12]. The Roza Canal in Washington State was one of the existing canal systems where hydrokinetic energy development was supported by the Energy Department of the United States [13]. British Waterways published a paper on proposals to utilize the 3541km of canals and rivers in Britain, and construct 25 small hydroelectric projects with a combined 40MW of generating capacity, through which 40,000 homes would be supplied with the sufficient P [14]. Botto et al. [15] examined a database of irrigation canals in the Piemonte region (north-western Italy) for mapping the resources for producing energy. They considered the application of hydrokinetic turbine technology to small and medium-sized channels to be a breakthrough in the generation of clean energy. In Taiwan [16] and the Lao People's Democratic Republic [17], irrigation water from agricultural canals has been used to power micro-HP installations. In general, streams with enough velocities are suitable for water current hydrokinetic generating. Additionally, there is a greater possibility of producing electricity the quicker the water runs.

Water leaving the draft tube (DT) or restrained flows or compensating flows at the base of tidal/hydro-power dams still contain a significant amount of hydrokinetic energy [18,19]. Reduced Q and reduced erosion on the shoreline's downstream hydraulic structures would result from using these flows to produce energy. Numerical methodology using Computational fluid dynamics (CFD) was used by Rozumalski and Fullarton [20] in order to replicate the Q in the Kansas Milford Dam. The findings suggested that downstream currents might move at speeds of 1.4–1.7 m/s. A statistical flow-field representation of Wanapum Dam's tailrace zone in southeast Washington State offered useful reference points and flow vector dispersion for hydro unit siting [21]. The HP project uses cooling water from the coal power plant to power a Kaplan turbine and generator to produce electricity in addition to using the energy from the downstream flows.

To effectively utilize ULH water energy, a suitable turbine must be chosen. Conventional turbines are categorized into two major forms Impulse and reaction. Pelton turbines, Turgo turbines, cross-flow turbines, Francis turbines, Kaplan turbines, and tubular turbines are the primary types of conventional HP turbines [4]. Additionally, there are around 20 different new hydrokinetic turbine types for the conversion of current energy including the conventional axial rotor, Savonius turbine, Darrieus turbine, Gorlov turbine, etc. [[22], [23], [24]] These newly developed hydrokinetic turbines can be divided into the lift and drag kinds based on the blade's hydraulic force or, for the horizontal and vertical axis turbines, the connection between the direction of fluid flow and the spinning axis [5].

Francis turbines with open flumes are often not employed in ULH applications. Francis turbines' tiny size and numerous blades make them difficult and expensive to machine at discharge rates lower than $1\;m^{3}/s$. Under the same ULH conditions, Francis turbines perform poorly in terms of flow capacity and PO when compared to Kaplan turbines [8]. Because the runner (RV) blades and guide vanes (GV) of Kaplan turbines are both changeable, they also operate more effectively over a wide range of conditions. The blade adjustment mechanism, however, is intricate and needs enough room in the RV hub for installation. As a result of their complexity and cost, Kaplan units are therefore more appropriate for scenarios requiring higher Q with lower hydraulic H. Fixed-blade propeller turbines are more suitable for ULH applications than Kaplan turbines and are far more cost-effective; however, their operating ranges for optimal power are limited [8].

Under the ULH circumstance and high Q, a tubular turbine is an excellent choice since they have low hydraulic losses because the water flows directly through it. To simplify systems and cut system costs, single- or non-arranged tubular turbines can be utilized in place of double-regulated ones at locations with lower total flow capacities. In order to lower the cost of producing Kaplan and tubular turbines, it is also possible to reasonably simplify the vane's shape and number [25]. As a novel axial-flow hydraulic turbine, a very low-head turbine has recently been shown to be acceptable for operation under ULH circumstances [26].

In order to accommodate low-head situations (i.e., 1−10m) and wide discharge ranges (i.e., 0.1−15 $m^{3}/s$), a new series of Archimedes screw turbines has been developed. Archimedes screw turbines have a modest impact on the environment, a slow rotational speed, and only minimal structural complexity [27]. They may also be fish-friendly. Using a multi-criteria analysis technique, Mumtaz et al. [28] consider the Archimedes screw to be the best-suited turbine for low-head HP. However, because of their size in terms of volume, Archimedes screw turbines are less practical for installation without constructing dams.

It can be concluded from the literature, that advancements in turbine technologies, optimized civil construction methods, and reduced project costs have rendered ULH HP an attractive, sustainable, and renewable energy source. Its proximity to human activity and perceived ecological friendliness contribute to widespread adoption. Key factors illustrating minimal environmental impact include unobstructed fish migration and navigation due to the absence of dams, as well as guaranteed downstream water flow facilitated by wide blade channels and low rotation speed. Despite the advantages, the systematic numerical investigations of the ULH turbines are missing from the literature. The literature lacks the comparative capacity and design analysis of the various ULH turbines so that the most feasible turbine design can be opted for maximizing renewable energy generation. In the current study, a few selected ULH turbine designs are numerically tested at a selected industrial water channel. Four turbine designs are presented, namely; a screw turbine, a Savonius turbine, an Axial turbine, and a Gate turbine. In the comparison, due to the nature of the turbine installation site bunki type and other pressure recovery system turbines are excluded in the current investigation. The turbines are studied using CFD tools to predict their performance in the selected site. The turbine that meets the target PO with the highest efficiency (η) is chosen for installation. The turbine performance along with the internal flow physics are presented in this study.

## 2 Description of the models

### 2.1 Channel dimensions

**Fig. 1:**
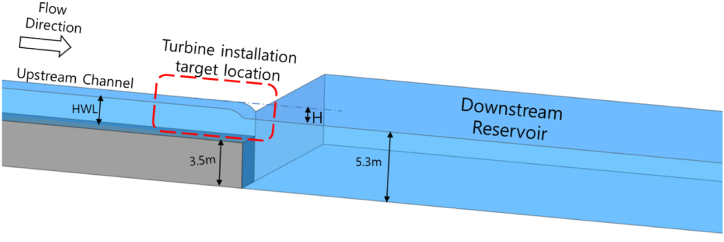
Target turbine installation site.

Among the ULH water sources, industrial waterways possess a high potential for installing ULH systems. The administrative approvals required for installing ULH in such sites are lower than installing at public rivers and ocean/marine water channels. Firstly, the four turbine designs are tested using CFD based on the flow conditions of a privately owned industrial water channel. Then the selected turbine would be manufactured and tested at the proposed site. The successful turbine design along with the test results would then be subject to approval for deployment in marine channels. The preliminary study to select a suitable turbine is presented here. The site selected for this study is the cooling tower outlet water canal of a CHP plant. The target installation site is shown in Fig. 1. The outlet water from the cooling tower is collected in the upstream channel and it flows into the downstream reservoir. After being pumped into the condenser, the water from the reservoir is then used to operate the cooling tower. The turbine is intended to be installed at the target location marked in the red box. The natural H available in the flow is 30 cm and thus hydrokinetic turbines are studied according to this condition. The downstream channel is 6 m deep with 5.3 m of minimum water level maintained for the cooling tower's secure functioning. The fluid flowing through the channel has a velocity of 1 m/s.

### 2.2 Different turbine models studied

Four different turbine models are studied and their configurations are explained here.

#### 2.2.1 Screw turbine concept

**Fig. 2:**
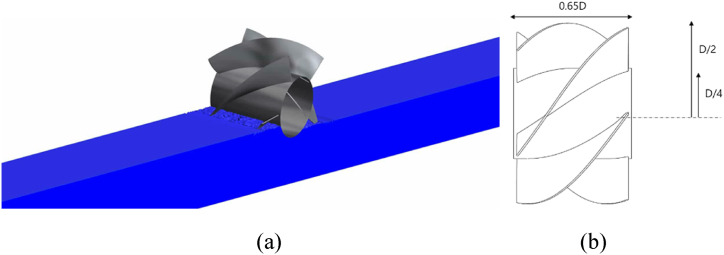
Horizontal-axis Screw turbine (a) concept, (b) dimensions.

The screw turbine is a hydrokinetic turbine concept inspired by the twisted blades of the Archimedes Screw Turbine as mentioned in the literature. However, a horizontal-axis screw-shaped turbine is tested here which is placed horizontally against the incoming flow as shown in Fig. 2. The concept of the screw turbine is illustrated in Fig. 2(a) and dimensions are presented in Fig. 2(b). The horizontal configuration of the screw turbine omits the requirement of dam construction as compared to a traditional Archimedes screw turbine. A five-bladed turbine on a central hub is placed on the surface of the water and connected to a generator using a belt-gear system. The specifications and dimensions are tabulated in Table 1.

**Table 1:** Screw turbine specification.

Parameter	Value	Description
Ro	D/2	Radius of tip
Ri	D/4	Radius of Hub
N	5	Blade's number
m	0.28	Helix turns
Ri/R0	0.5	Hub-to-tip ratio
t	4mm	Thickness of blade
L	0.65D	Length of the turbine
S	1.9812	Pitch
C	3.81m	Length of cord
S/C	0.52	Pitch-Cord ratio

The turbine is partially submerged (by 30 %) and uses the water's kinetic energy (KE) in motion to generate electricity. The submergence depth of the turbine is determined from a parametric study conducted in a previous publication [29]. The hub-to-shroud ratio and the submergence depths are two of the design factors of the parametric study used in the prior study. The submergence depths were varied from 20 % to 50 %, and the hub-to-shroud ratio was varied from 0.3 to 0.7. It was found that the maximum power production was achieved when the hub-to-shroud ratio was set to 0.5 and the device was submerged in water to a depth of 30 %.

#### 2.2.2 Savonius (horizontal and vertical) turbine

**Fig. 3:**
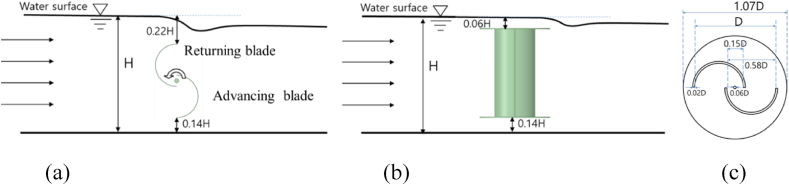
Savonius turbine models (a) horizontal orientation, (b) vertical orientation, and (c) dimensions.

The Savonius turbine is a familiar concept since they are widely used in wind power extraction. However, recently growing interest showed the application of Savonius turbines as a hydrokinetic turbine for generating HP [30,31]. The turbine geometry for this particular site is designed by considering several geometrical parameters obtained from literature and the length of the horizontal turbine is determined according to the channel width [[32], [33], [34], [35], [36], [37], [38]]. Two installation configurations are tested using the same turbines. Firstly, the turbine is tested in a horizontal configuration similar to the screw turbine installation but completely buried in water as shown in Fig. 3(a). Then the turbine is tested vertically immersed in the water as shown in Fig. 3(b). The RV diameter is 1.5 m with a blade thickness of 0.03 m. The dimensions are given in Fig. 3(c). One important factor that can affect the Savonius turbine's performance is the distance between the turbine and the channel bottom. This effect in hydro Savonius turbine has been studied by Katayama et al. [33] by experimental methods and concluded that the suitable distance between the channel bottom and turbine RV is between 0.1 and 0.2 times the RV diameter. Therefore, a similar value is chosen in the present study presented in terms of H.

#### 2.2.3 Axial turbine (tubular and ducted) concepts

**Fig. 4:**
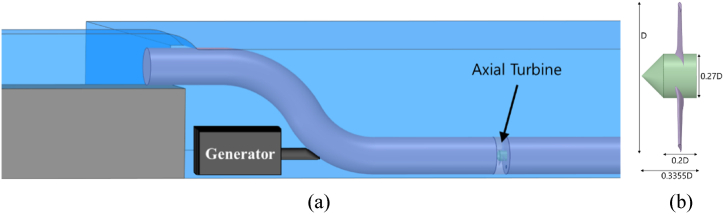
Tubular Axial turbine (a) concept, (b) turbine dimensions.

The axial turbine is a 3-bladed windmill-like turbine usually used in marine/ocean applications [[39], [40], [41], [42], [43], [44], [45]]. The axial turbine is tested in two configurations namely, tubular axial turbine and ducted axial turbine. The tubular turbine consists of a large tube of diameter 1.4 m placed at the exit of the upstream channel to collect water into the tube. The KE of the water entering the tube is transformed into mechanical energy (ME) by the axial turbine. The remaining water simply overflows the tube into the reservoir thereby submerging the tubular turbine system. The entire upstream Q is not input into the turbine in order to avoid the risk of overflowing which may hinder the cooling tower's secure functioning. The axial turbine is installed inside the tube after the S-shaped pipe bends such that the flow is fully developed before reaching the turbine as depicted in Fig. 4(a). The generator would be submerged below the S-bend. The dimensions of the turbine are given in Fig. 4 (b).

**Fig. 5:**
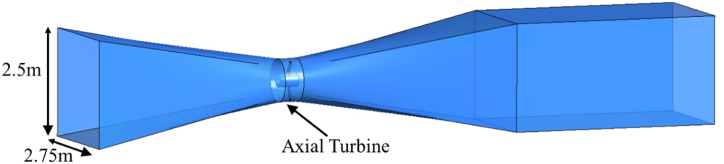
Ducted Axial turbine concept.

The ducted axial turbine is an idea similar to the tubular turbine but the turbine RV is placed inside a duct with convergent and divergent designs as shown in Fig. 5. The convergent duct gathers a sizable amount of the incoming flow. The fluid velocity increases as the duct converges towards the turbine, transferring maximum KE to the RV blades. The RV converts the velocity of the fluid into ME. A generator system converts the rotating energy into electricity. The ducts are mounted on several support structures which are not shown here.

#### 2.2.4 Gate turbine concept

**Fig. 6:**
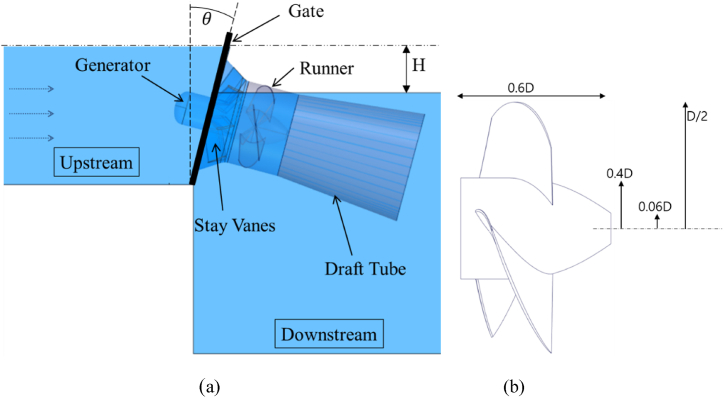
Gate turbine (a) components and operating principle, (b) basic dimensions.

The gate turbine is an axial Kaplan-type turbine mounted on a movable gate structure. The turbine consists of a gate structure, DT, RV and stay vane (SV). The generator is installed inside a submerged SV hub as shown in Fig. 6(a). The RV profiles are designed similarly to an axial Kaplan turbine but with a fixed blade angle. The SV consists of 7 vanes and the RV consists of 4 blades. The basic dimensions of the turbine are given in Fig. 6(b) in terms of RV diameter. The water through the upstream passage is held by the gate structure acting to maintain the upstream fluid level. The gate angle can be adjusted to an angle θ to control the water level. The water is then directed into the turbine through the stay vanes. The KE of the fluid passing the turbine is converted to ME by the rotation of the RV. The generator converts the ME of the RV into electrical energy. The H of the turbine is determined by measuring the difference in water levels between the upstream and downstream areas. Since an artificial H is created, the gate turbine may not fall under the class of hydrokinetic turbines. It is calculated that the gate turbine has a specific speed of 670.

## 3 Numerical model

### 3.1 Governing equations

The mass and momentum conservation are the formulas that govern fluid transportation. Many hydraulic flows in engineering applications can be regarded as incompressible. Additionally, it is believed that fluid is Newtonian, and the flow in the current application can be thought of as isothermal. The viscosity of an isothermal, incompressible flow is constant. Combining all these conditions, the continuity and momentum equations can be written as [46];

(1) $$\frac{\partial u_{i}}{\partial x_{i}}=0$$

(2) $$\rho \frac{\partial u_{i}}{\partial t}+\rho u_{j}\frac{\partial u_{i}}{\partial x_{j}}=-\frac{\partial p}{\partial x_{i}}+\frac{\partial }{\partial x_{j}}\left[\mu \left(\frac{\partial u_{i}}{\partial x_{j}}+\frac{\partial u_{j}}{\partial x_{i}}\right)\right]+\rho g_{i}$$

Eqs. (1), (2) are called the Navier-Stokes (NS) equations and the unknown terms can be found in reference [46]. The present investigation uses ANSYS-CFX software for the numerical calculation which employs Reynolds-Averaged NS (RANS) equations to formulate the NS equations in terms of velocity $u_{i}(x_{i},t$) and pressure $p\left(x_{i},t\right)$ for lesser computing requirements than a direct solution.

Steady-state single-phase (water) simulations are used to represent the fully submerged turbine models (savonious, axial and gate) except for the screw turbine. For the screw turbine model, the turbine is partially submerged in water which calls for a multiphase simulation with air at the top and water at the bottom of the computational domain. The continuity and momentum equations for the transient multi-phase model are given in the below eqs. (3), (4) [46]:

(3) $$\frac{\partial \rho_{m}}{\partial t}+\frac{\partial \rho_{m}{\bar{u}}_{i}}{\partial x_{i}}=0$$

(4) $$\frac{\partial \rho_{m}\bar{u_{i}}}{\partial t}+\frac{\partial \rho_{m}{\bar{u}}_{i}{\bar{u}}_{j}}{\partial x_{j}}=-\frac{\partial \bar{p}}{\partial x_{j}}+\frac{\partial }{\partial x_{j}}\left[\mu_{m}\left(\frac{\partial {\bar{u}}_{i}}{\partial x_{j}}+\frac{\partial {\bar{u}}_{j}}{\partial x_{i}}\right)\right]-\frac{\partial }{\partial x_{j}}\tau_{ij}^{RANS}$$

Where $\rho_{m}$ is the mixture (two-phase) density ($kg/m^{3}$), $\mu_{m}$ is the mixture dynamic viscosity of the fluid (Pa.s), $\bar{u},u^{\prime }$ and $\bar{p}$ represents ensemble-averaged velocity (m/s), fluctuation component of the velocity (m/s), and averaged pressure of the flow (Pa) respectively. The indicial notations i and j depict the cartesian coordinate frame. The averaging of NS equations results in an unknown term known as Reynolds stress tensor $\tau_{ij}^{RANS}=\rho \bar{u_{i}^{\prime }u_{j}^{\prime }}$ which formulates six extra unknowns that need to be modelled.

### 3.2 Turbulence model

The shear stress transport (SST) model is utilized in the current investigation to address the turbulent flow behaviour through the turbines. Several researchers employed the SST model to investigate the complex flows inside the turbomachinery and suggested that the model predicts the flow characteristics accurately [47,48]. The SST model, created by Menter [49], is a mixing of the k−ϵ model at the surface and the k−ω model in the outer area. By utilizing a blending function, the two models have been combined. The SST k−ω model equations for the two-phase flow may be expressed in eqs. (5), (6) by multiplying the k−ω model equations by the function $F_{1}$ and the altered k−ϵ model equations by $1-F_{1}$.

(5) $$\left[\frac{\partial \rho_{m}k}{\partial t}+\frac{\partial \rho_{m}\bar{u_{j}}k}{\partial x_{j}}\right]=\frac{\partial }{\partial x_{j}}\left[\left(\mu_{m}+\frac{\mu_{t^{m}}}{\sigma_{kSST}}\right)\frac{\partial k}{\partial x_{j}}\right]+P_{k}-\beta^{\prime }\rho_{m}k\omega$$

(6) $$\left[\frac{\partial \rho_{m}\omega }{\partial t}+\frac{\partial \rho_{m}\bar{u_{j}}\omega }{\partial x_{j}}\right]=\frac{\partial }{\partial x_{j}}\left[\left(\mu_{m}+\frac{\mu_{t}^{m}}{\sigma_{\omega SST}}\right)\frac{\partial \omega }{\partial x_{j}}\right]+\left(1-F_{1}\right)\frac{2\rho_{m}}{\sigma_{\omega k-\epsilon }\omega }\frac{\partial k}{\partial x_{j}}\frac{\partial \omega }{\partial x_{j}}+\frac{\alpha_{SST}\omega P_{k}}{k}-\beta_{SST}\rho_{m}\omega^{2}$$

These equations' closure coefficients ($C_{SST}$) are all given in terms of the blending function which is presented in eq. (7).

(7) $$C_{SST}=F_{1}\left(C_{k-\omega }\right)+\left(1-F_{1}\right)C_{k-\epsilon }$$

By including a limiter into the formulation of mixture eddy viscosity ($\mu_{t}^{m}$), the appropriate transport behaviour may be established in eq. (8);

(8) $$\mu_{t}^{m}=\frac{\rho_{m}a_{1}k_{m}}{\max\;\left(a_{1}\omega_{m},S_{m}F_{2}\right)}$$

Because the underlying assumptions are incorrect for free shear flows, the limiter is restricted to the wall boundary layer by the blending function $F_{2}$. $S_{m}$ is the strain rate magnitude. Tangent hyperbolic functions of numerous flow variables, such as $k_{m}$, $\omega_{m}$, and their spatial derivatives, are used to express both $F_{1}$ and $F_{2}$. The mixture density and dynamic viscosity can be expressed in eqs. (9), (10);

(9) $$\rho_{m}=\rho_{v}\alpha_{v}+\rho_{l}\left(1-\alpha_{v}\right)$$

(10) $$\mu_{m}=\mu_{v}\alpha_{v}+\mu_{l}\left(1-\alpha_{v}\right)$$

Where vapour, liquid, mixture and vapour volume fraction are represented by *v*, *l*, *m*, and $\alpha_{v}$ respectively.

### 3.3 Boundary conditions and numerical procedure

The performances of the turbines are studied by solving the governing and turbulence equations using the numerical solver. The turbine components and their interfaces are combined to form the fluid domains of the turbine. The computational domain for the kinetic turbines consists of the 6 planes of parallelepiped. The Screw turbine is studied using a multiphase, free-surface, transient setting by incorporating air and water in the domains. For the rotor-stator interaction, the transient model uses the interface between spinning and stationary fluid domains. In simulating unsteady open channel flow, the dynamics of air and water are modelled separately, considering their different densities and interactions. The exchange between these two phases is addressed using a standard free surface model, incorporating buoyancy and drag forces. To account for momentum transfer, a standard interface drag force coefficient of 0.44 is used. Despite the complexity, a stable computation is achieved through the application of a model of homogenous turbulence for both fluids in the inhomogeneous multiphase simulation. At the interconnection between the immersed turbine and the channel, a transient rotor-stator interface is implemented. Hydrostatic pressure for water and ambient pressure for air are the initial conditions. This arrangement guarantees that the water pressure rises with depth as a result of gravity. One meter per second is the maximum speed allowed at the input, and there is a hydrostatic pressure distribution opening at the outflow. Using a step function, the volume of fluid (VoF) throughout the domains is defined. In particular, this step function applied to the water's VoF yields the depth of the upstream water. In terms of mathematics, the VoF is defined in eq. (11);

(11) vofWaterUp=step(0[m]−y(1[m]))

**Fig. 7:**
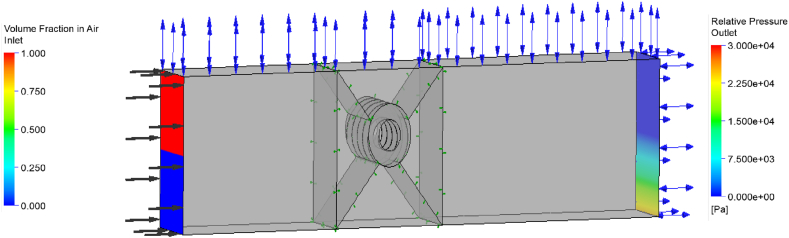
Computational domain of the screw turbine.

Where the direction of gravity is denoted as -y, any position below 0 m is considered submerged in water, particularly relevant for a turbine submerged to 50 %. The VoF representing air upstream is determined as '1-vofWaterUp'. A comparable formula is applied to calculate the depth of water downstream. Using a step function, the water level is adjusted to account for both low- and no-head situations. The computation domain and boundary condition for the screw turbine is presented in Fig. 7 and Table 2. With ambient pressure at the top and hydrostatic pressure at the outlet, the top surface and the outlet are both classified as apertures. The air inlet volume fraction is shown in red, while the water input volume % is shown in blue. The deeper the water is, the higher the hydrostatic pressure at the exit. The turbine rotates counterclockwise as seen from the side. The turbine's revolutions per minute (rpm) are used to calculate the timestep and overall duration for the unstable simulations. A steady output is often reached by the turbine after five to eight rotations.

**Table 2:** Boundary conditions for the unsteady scheme of screw turbine.

Parameter		Value	Parameter		Value
Domain Initialization	Pressure	Hydrostatic pressure	Turbulence model	Air	SST
	Air	U, V, W = 0 m/s VoF = 1 for + y		Water	SST
	Water	U = Inlet velocity V,W = 0 m/s VoF = 1 for -y	Surface Tension Coefficient		0.072 N/m
Buoyancy Model	Gravity	Y = −9.81 m/s	Interphase transfer		Free surface
	Ref. density	1.185 kg/m^3^	Drag coefficient		0.44
Fluid buoyancy model		Density difference	Analysis type		Unsteady
Multiphase model		Inhomogeneous			

**Fig. 8:**
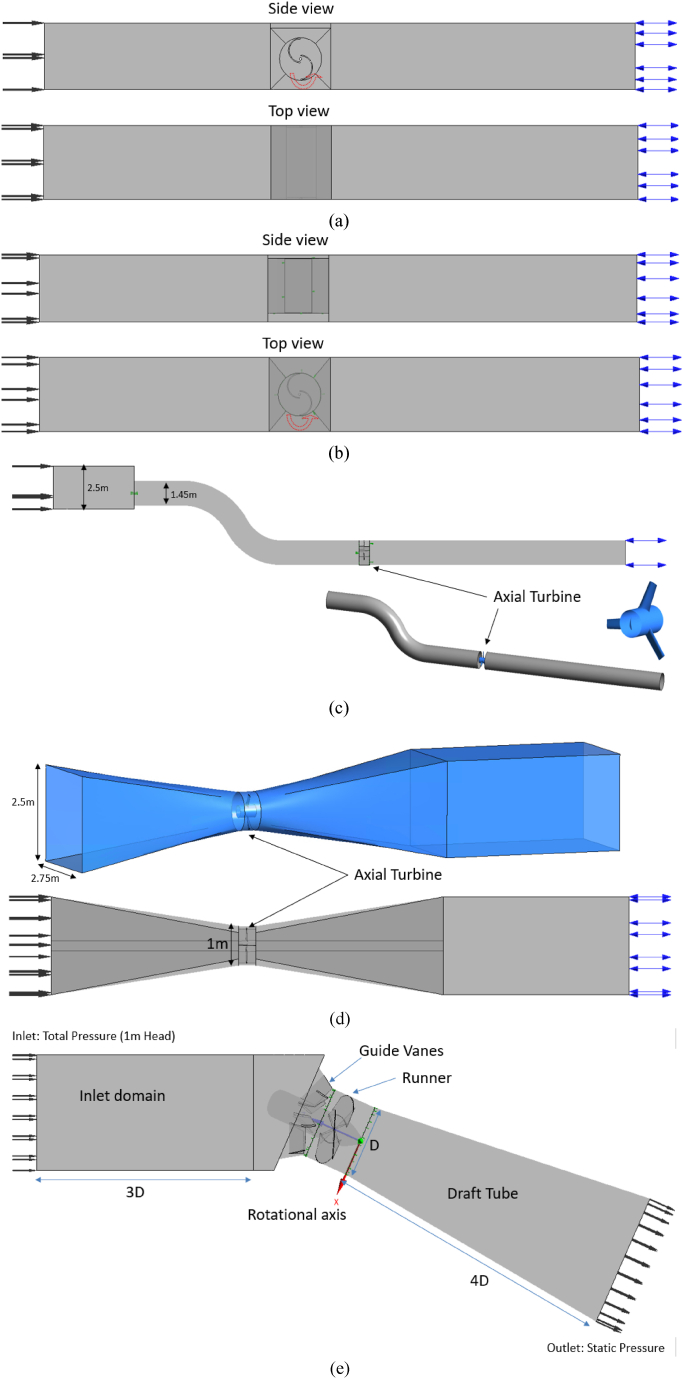
Computational domains (a) Horizontal Savonious turbine (b) Vertical Savonious turbine (c) Tubular axial turbine (d) Ducted axial turbine (e) Gate turbine.

The Savonius turbines, the axial turbines, and the gate turbines being submerged in water are modelled using steady-state single-phase (water) simulations. Every other turbine component—aside from the RV—is classified as a stationary domain. The General Grid Interface (GGI) technique is employed for data transfer between rotating and stationary domain boundaries. In steady computations, the interface between rotational and stationary fluid domains is called the stage (mixing plane). For all the simulations, the inlet and outlet boundaries are defined according to the site conditions. Generally, a velocity inlet is assigned at the upstream channel inlet. An automated wall function and a no-slip boundary condition are used on the walls of the computational domains. The computational domains of the kinetic turbines which undergo the steady state analysis are depicted in Fig. 8. Fig. 8(a) represents the side and top view of the horizontal Savonious turbine and Fig. 8(b) depicts the side and top view of vertical Savonious turbine. The computational domains of tubular axial turbine, ducted axial turbine and gate turbine are presented in Fig. 8(c), (d) and (e), respectively. The boundary conditions for these turbines are tabulated in Table 3.

**Table 3:** Boundary conditions for turbines went through steady-state analysis.

Parameter		Value
Domain Initialization	Pressure	0
	Velocity	Inlet velocity
Inlet Velocity		1.0545 m/s
Outlet Pressure		0 Pa
Turbulence model		SST
Interface		Stage
Analysis type		Steady

First-order turbulence numerics are used, and high-resolution and second-order central techniques are used to discretize the convection and diffusion terms, respectively. An upwind differencing method with an anti-diffusive correction is used by the high-resolution system. The Root Mean Square (RMS) value of the residuals, which is fixed at $10^{-6}$ is used for the time-dependent convergence criteria. An appropriate time step is essential in transient CFD simulations. Generally speaking, the time step (Δt) needs to be low sufficing to record the quick-moving characteristics that take place in the hydraulic machinery [50,51]. Therefore, in the current investigation, the unsteady responses were estimated using a Δt based on eq. (12) where N corresponds to the turbine rotational angle.

(12) $$\Delta t=\frac{Step\;angle\times 60}{N\times 360}$$

### 3.4 Mesh generation and independency

Grid independence is verified on the numerical grid which is created for the fluid domains. Because of the complex design, tetrahedral meshing is used for all stationary domains. While the RV blades of the Savonius and Screw turbines are created using the ANSYS meshing tool, the RV blades of the axial and gate turbines are meshed using Turbogrid. Prism mesh was layered multiple times at the RV surfaces in order to resolve the boundary layers. More consideration was given to meeting the orthogonal quality and aspect ratio requirements because the intricate flows within the flow domain are highly influenced by the grid quality.

Grid independence tests were performed using steady-state calculations to determine the optimal grid and minimize the effect of mesh density on the numerical solution. For a grid convergence inquiry, the most reliable method is the Grid Convergence Index (GCI), which was created based on the Richardson extrapolation approach [52]. For three different sets of grids with meaningful resolutions, a fine grid convergence index ($GCi_{fine}$), a key variable (ϕ), and an approximation relative error ($e_{a}$) were investigated based on eq. (13). The PO is the main variable in the simulation.

(13) $$e_{a}=\left|\frac{\phi_{1}-\phi_{2}}{\phi_{1}}\right|$$

where, respectively, suffixes 1 and 2 denote new and old mesh. The Grid convergence index is expressed as follows in eq. (14);

(14) $${GCI}_{fine}=\frac{1.25e_{a}}{r-1}$$

**Fig. 9:**
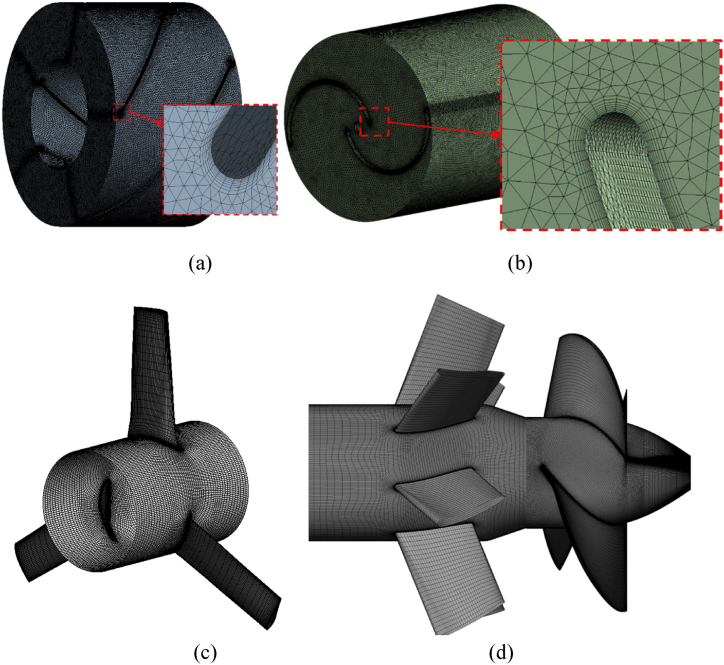
Mesh generated for the turbine designs (a) Screw Turbine (b) Savonius turbine (c) Axial turbine (d) Gate turbine.

For further detailing one can refer to Ref. [52]. The anticipated discretization errors for each of the three meshes are shown in Table 4. The fine, medium, and coarse mesh nodes are denoted by the integers $N_{1}$, $N_{2}$, and $N_{3}$. Relatively little approximate and extrapolated relative error characterizes the resulting ${GCI}_{fine}$ and is less than 1 % for the screw turbine and the gate turbine. The grids for other turbine models are generated based on these results. In Fig. 9, the grid images are shown. The meshes generated on flow domains of the screw turbine, Savonius turbine axial turbine and gate turbine are depicted in Fig. 9(a), (b), (c) and (d), respectively. The average wall $y^{+}$ value is kept below 30.

**Table 4:** GCI test for the Screw turbine and the Gate turbine.

Parameter	Screw Turbine	Gate Turbine
N1, N2, N3	2,335,753; 912,545; 409,168	2,987,407; 1,706,207; 702,367
r_21_	1.368	1.32
r_32_	1.307	1.56
P	4.1822	1.894
GCI^21^_fine_	0.323	0.15
GCI^32^_medium_	0.046	0.26

## 4 Results and discussion

### 4.1 Validation of the numerical model

The validation of the numerical model is conducted to ensure that the outcomes from the CFD results are reliable and accurate. Validation is conducted for the basic steady-state CFD methodology which acts as a basis for all the turbine models presented in this study. For the purpose of validation, a low-head axial hydraulic turbine geometry developed by Park et al. [53] is secured and numerical domains are generated based on the experimental conditions from the literature. Their axial turbine model is the primary basis for the gate turbine design presented in this paper. There were several phases to the development process. First, by creating a CFD model that precisely matched the experiment's dimensions and boundary conditions, Park et al.'s experiment results [53] were utilized to validate the CFD methodology. According to the experiment, the H condition was 4.2 m, the rotational speed was 600 rpm, and the Q was 1 $m^{3}/s$. Following the validation of the CFD methodologies, the turbine was scaled up to the current site circumstances using the retrieved DT shape, RV profiles, and SV profiles. To the turbine, an extra movable gate component is added with the intention of creating an artificial H. By creating a gravitational potential H, the gate structure assists in converting a no-head site into an ultra-low head site. The first gate turbine design, complete with all of its parts, is tested in the following stage using a CFD model to examine its initial performance.

**Fig. 10:**
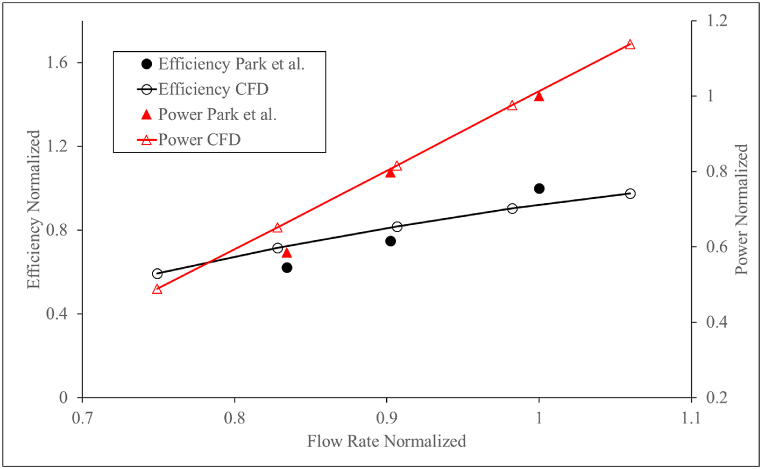
Numerical validation with Park et al. [53].

The numerical methods, governing equations, turbulence equations, and mesh generation elaborated in section 3 are used in the CFD model. The validation process involved comparing various hydraulic characteristics, such as η, PO, and H between the CFD simulations and the experimental results. The validation results are shown in Fig. 10. The agreement between the two sets of data was found to be reasonable with an error of less than 5 %, indicating that the CFD model was able to accurately capture the flow behaviour in the system. The same CFD setting is used to create the numerical conditions of the other turbine models mentioned in this paper. The basic methodology of the CFD models including the continuity equations, transport equations, and turbulence equations remain the same for all the turbine models with the steady-state CFD model with only changes in the boundary conditions, rotating speed and the rotating angle. Therefore, validating one type of turbine is considered to be sufficient to create CFD models for other turbine designs.

### 4.2 Hydraulic performance of the hydrokinetic turbines

This section presents the hydraulic performance of the turbines as determined by the CFD simulations. The Tip Speed Ratio (TSR) and Coefficient of Power ($C_{p}$) are the metrics used to show how well hydrokinetic turbines work. The ratio of the free stream flow velocity to the tangential velocity at the blade tip is known as the TSR. The energy recovered by the turbine divided by the water's KE is called $C_{p}$. Mathematically the parameters are represented by eqs. (15), (16);

(15) $$TSR=\frac{r\omega }{V}$$

(16) $$C_{p}=\frac{\tau \omega }{\frac{1}{2}\rho AV^{3}}$$

where A is the swept area of the water-submerged blade, τ is the torque produced by the RV, V is the turbine input velocity, r is the turbine's radius, and ω is the angular velocity.

**Fig. 11:**
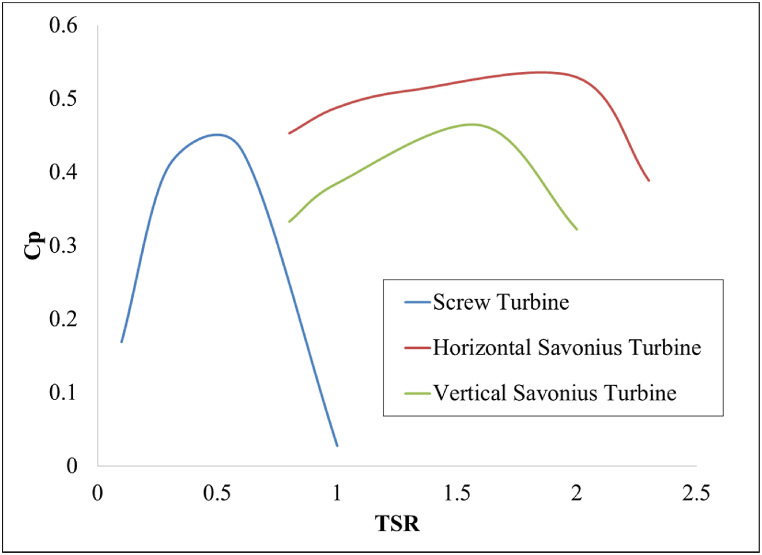
TSR test of hydrokinetic turbines.

The hydrokinetic turbine performance is tested at various tip speed ratios similar to the wind turbines. The tip speed ratios are obtained by testing the hydrokinetic turbines at various rotational speeds. The RPMs were tested in an increasing order starting from 1 rpm up until the $C_{p}$ value produced a negative value. Further increasing the RPM produces a negative $C_{p}$ value which is incorrect since the turbine is forced to rotate manually rather than allowed to be rotated by the force of the incoming water. The TSR values are calculated from these RPMs. The TSR vs $C_{p}$ curve gives us a clear indicator of the performance of the turbine models. The performance of the Screw turbine, the horizontal and vertical Savonius turbines are compared in Fig. 11. The TSR values of these turbines are observed to be between 0 and 2.5. These results are in synchronous with the chart projected by Arrieta et al. [54].

**Fig. 12:**
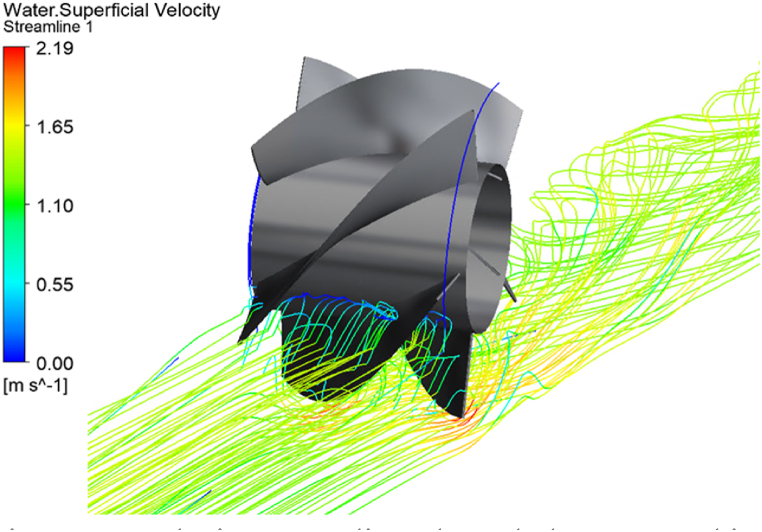
Velocity Streamlines through the screw turbine.

In the screw turbine model, a peak $C_{p}=0.43$ is obtained at a TSR=0.6. For all other TSR values, the $C_{p}$ drops below 0.3. The turbine operates in the TSR range of 0–1 and it can be said that the best efficiency point (BEP) of the screw turbine is achieved at 0.6 TSR. The flow through the screw turbine is shown in Fig. 12 by plotting the superficial velocity streamlines of water. The fluid loses its velocity upon impingement with the blade thereby converting its KE into ME to rotate the RV. The remaining water flows underneath the turbine at a high velocity.

**Fig. 13:**
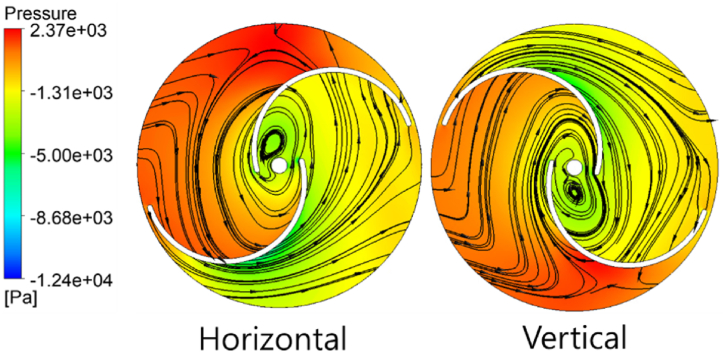
Pressure contour and velocity streamlines of Savonius turbine.

The vertical Savonius turbine has a peak $C_{p}=0.46$ at TSR=1.6. The operating range of this type of turbine is between 1 and 2 TSR. In the horizontal Savonius turbine, the performance is higher than its vertical counterpart. The peak $C_{p}$ value was obtained at 0.53, an increase of 15.2 % at a TSR value of 2. The pressure contour of both configurations is shown in Fig. 13. The side view of the horizontal turbine and top view of the vertical turbine are shown along with the velocity streamlines at the peak $C_{p}$ value. The horizontal arrangement revolves counter-clockwise, whereas the vertical turbine rotates in a clockwise manner. In the horizontal configuration, the RV blade which stands concave to the incoming flow is positioned at the bottom of the channel and the convex blade is at the top. As a result, the concave surface experiences higher hydrostatic pressure at the bottom of the channel creating a pressure difference between the two sides of the blade. The KE of the flowing water along with the pressure gradient across the RV contributes to a higher PO in the horizontal configuration. This does not apply to the vertical Savonius turbine since the hydrostatic pressure acts equally on both the RVs and the turbine rotates solely on the KE of the flowing water. Hence, the PO of the vertical Savonius turbine is lower than the horizontal configuration.

### 4.3 Hydraulic performance of the gate turbine

#### 4.3.1 Initial performance

The performance of the reaction-type turbine cannot be assessed by the TSR and $C_{p}$ parameters. They are evaluated based on their η, which is determined by using the following eq. (17) to determine the PO, H, and Q;

(17) $$\eta =\frac{\tau \omega }{\rho gHQ}$$

**Fig. 14:**
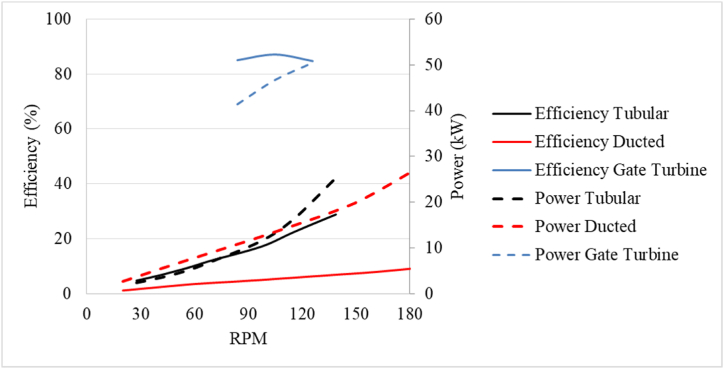
Hydraulic performance of the reaction turbines.

The axial turbines which are normally considered hydrokinetic turbines, are treated as reaction turbines since they are placed inside a pipe or a duct since the pressure drops inside the tube. The turbine performance of the axial turbines and the gate turbine at a 14° gate angle is plotted in Fig. 14. The turbines are tested for various rotational speeds (RPM).

The gate turbine showed the highest η at 87.07 % with a PO of 46.7 kW at a H of 0.9 m. The BEP is obtained at an rpm of 105. The η decreases at lower and higher rotational speeds. The PO increases as the rpm increases and the highest power is obtained at 126 rpm.

**Fig. 15:**
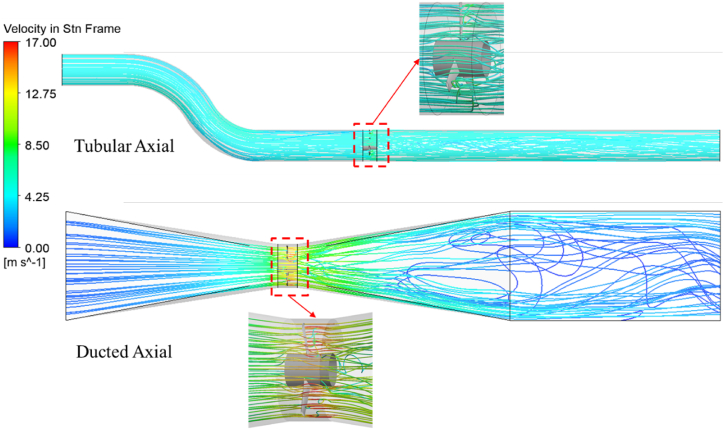
Velocity streamlines through the axial turbine configurations.

The Axial turbine is tested from a large range of RPMs ranging from 30 to 180 rpm at constant Q. Between the two types of axial turbines, the tubular axial turbine showed higher η and power than the ducted turbine. This is a result of the ducted axial turbine's venture effect, which causes a significant pressure differential between the domain's inlet and exit. The large pressure difference increased the H in the domain thereby decreasing the η of the ducted turbine. Fig. 15 shows the velocity streamlines through the axial turbine configurations.

**Fig. 16:**
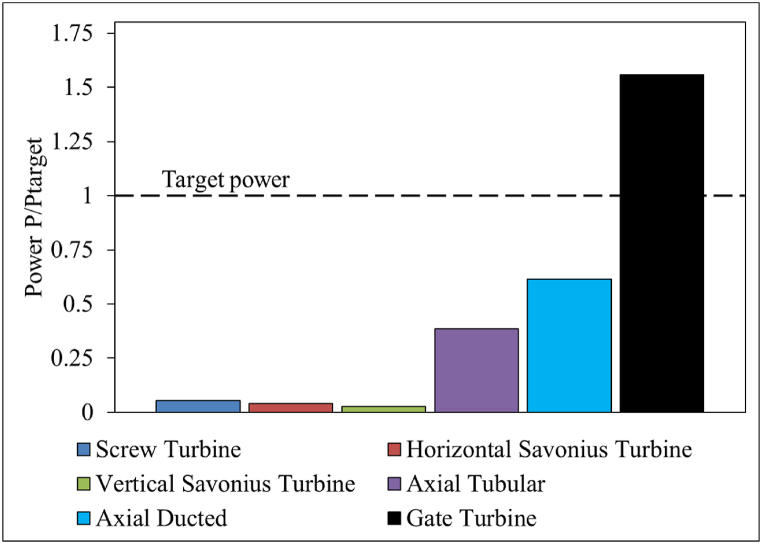
Performance comparison of the turbine models with the target power.

The PO at the design point of the turbine designs tested in this investigation is compared in Fig. 16. At this particular site, the Screw turbine and the Savonius turbine showed poor performance with very low PO. The ducted and tubular axial types showed higher PO than the drag-type models but failed to achieve the target PO. On the other hand, the Gate turbine showed promising results by meeting both the target PO and η. This is because the majority of the upstream Q was directed to flow through the turbine with the help of the gate structure. However, in a real case, some of the water may be allowed to overflow into the downstream reservoir above the gate structure to avoid damming the gate structure and maintaining the cooling tower's safe operating state.

#### 4.3.2 Parametric study of the gate turbine

**Fig. 17:**
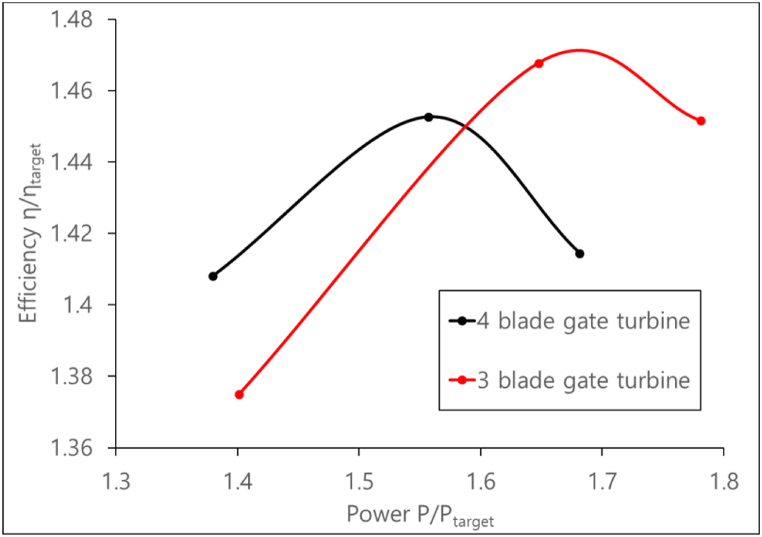
Performance of 3-blade and 4-blade gate turbine.

**Fig. 18:**
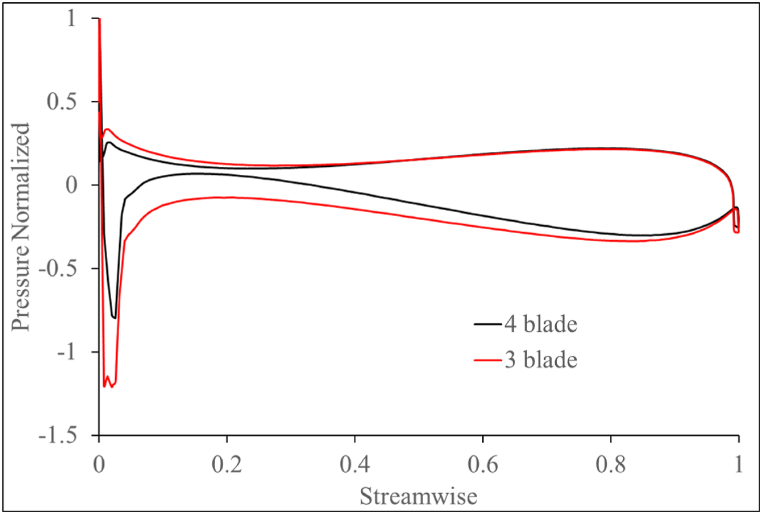
Blade loading distribution at the midspan of the gate turbine RV blades.

Testing is done on the gate turbine for 2 configurations of 3-blades and 4-blades; and at three gate angles namely, 14°, 24°, and 34°. The performance of the 3 and 4-bladed gate turbines are compared in Fig. 17. The η and P values are measured at different RPMs and are normalized by their target values. The 3-blade turbine shows a higher η with a higher PO than the 4-blade design. With a significantly lower pressure at the suction side, the pressure differential between the RVs' pressure sides and suction sides has grown. The blade loading pattern depicted in Fig. 18 illustrates this. The wider the area under the graph, the larger the pressure difference across the RV, and the larger the PO. The larger PO comes at the cost of a region of reduced pressure at the RV's leading edge (streamwise 0). If the zone of low pressure near the leading edge decreases further sharply, cavitation risk might arise in the turbine and is the scope for future studies. The 3-blade design's BEP is also shifted towards a higher power than the 4-blade design. This is due to the higher Q observed in the 3-blade design due to the increase in blade annular passage area of flow as a result of removing 1 blade.

**Fig. 19:**
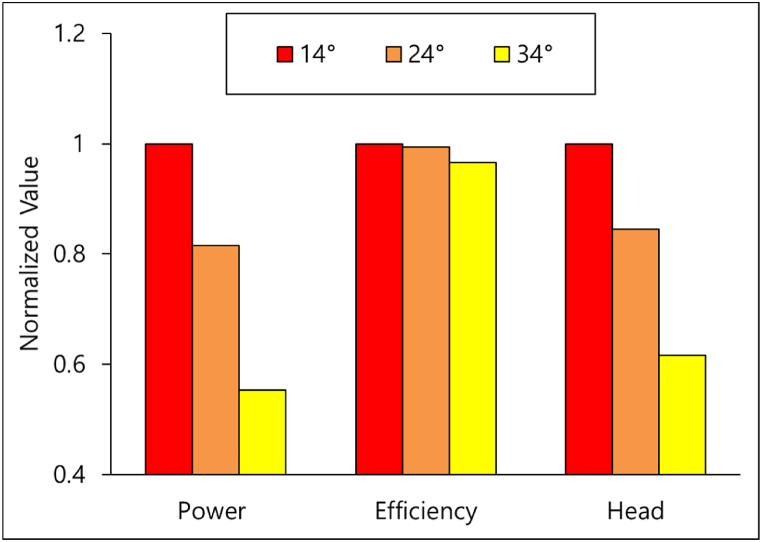
Performance of gate turbine at various gate angles.

**Fig. 20:**
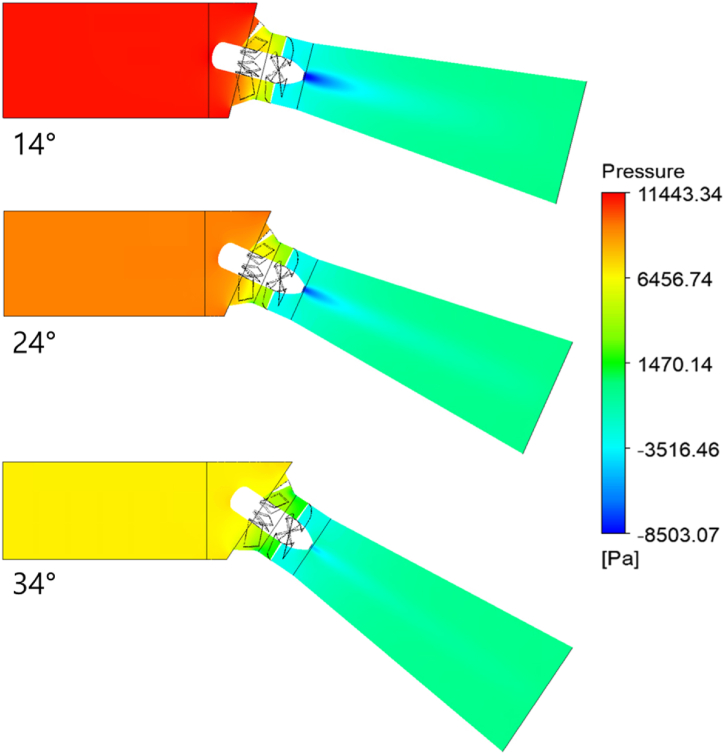
Pressure contour of the gate turbine at various gate inclination angles.

Since the 3-blade gate turbine design delivers a larger PO, it is selected for the gate inclination angle study. The hydraulic performance comparison of the three gate angles is illustrated in Fig. 19. The parameters are non-dimensionalized with their maximum values. It is seen that the P of the turbine decreases with an increase in the inclination angle. The P decreases by 18.48 % when the gate angle is increased from 14° to 24° and further decreases by 32.11 % when the angle is lowered from 24° to 34°. As the gate is more inclined, the overflow Q increases, thereby decreasing the Q through the turbine. Moreover, the H of the turbine also decreases with increasing gate angle which is an expected outcome. The pressure contours plotted in Fig. 20 show the reduction in pressure upstream due to the fall in the H. The less the upstream and downstream pressure differences, the lower the PO through the turbine. The cutback in H and Q, being the terms in the denominator of the η equation, is expected to boost the turbine η. However, the reduction in the PO (numerator) has offset this rise in η. Therefore, the η of the turbine at the 24° gate angle is reduced by only 0.63 % compared to the 14° gate angle. The η drops further by only 2.77 % when the gate angle is increased from 24° to 34°. In other words, it can be said that the P of the turbine can be controlled by adjusting the gate inclination angle according to the site flow conditions without a significant loss in the turbine η. This also grants a larger operating range for the turbine operation.

The 3-bladed Gate turbine installed at a 14° gate inclination angle is chosen as the final turbine model suitable for installation at the proposed site.

### 4.4 Feasibility on the environmental friendliness

The turbine selected from this study is designed to be used in the forebay of a CHP plant. Since CHP plants use purified water, it is an environment where aquatic life does not exist. Hence, in the present study, the environmental concerns were not addressed. However, for IGT, it can be applied not only to these specific sites but also to other environments in the future (e.g., natural rivers, and canals). Therefore, the current gate turbine design must be improved to be environmentally friendly. The following methods are considered to address this issue. Firstly, a grid-like filter may be installed at the entrance of the IGT to prevent foreign substances and aquatic life from entering the turbine. This is a matter that is being considered and discussed in practice before being applied to the actual site. Secondly, a detour for aquatic life (fish passage) may be considered when installing in a river with aquatic life. In fact, this is a general consideration when the hydraulic turbine for small HP is applied to rivers or streams. Hydraulic power generation is an economically superior renewable energy generation method except for the initial installation cost. It is also known to have less impact on environmental destruction than traditional fossil power generation. Nevertheless, the impact on aquatic life and ecosystem problems is constantly being discussed by many environmentalists. Therefore, it is agreed that the final prototype of the turbine must incorporate these techniques to achieve a fish-friendly turbine. This is currently under development.

## 5 Conclusion

ULH turbine technologies have become imperative for harnessing electricity from previously overlooked low-head renewable energy sources. In this study, various ULH turbine technologies were examined for their viability in exploiting these resources. Specifically, screw, Savonius, axial, and Gate turbine designs were evaluated for installation in the cold-water basin of an industrial cooling tower. The screw turbine was partially submerged by 30 %, while the Savonius turbine was fully submerged, with both horizontal and vertical configurations investigated. Additionally, the axial turbine was studied in tubular and ducted configurations, while the Gate turbine underwent testing with both 3-blade and 4-blade designs. Further analysis focused on the 3-blade Gate turbine, examining its performance at varying gate inclination angles of 14°, 24°, and 34°. The key findings from the numerical study are outlined as.

• The screw turbine exhibited the lowest performance, achieving a peak C_p value of 0.43 at a TSR of 0.6. In comparison, the Savonius turbine displayed enhanced performance, with increases in C_p values of approximately 23.25 % and 7 % for horizontal and vertical configurations, respectively.
• The horizontal Savonius turbine outperformed its vertical counterpart, demonstrating a 15.2 % increase in C_p. The peak C_p value was attained at a TSR ratio of 2.
• The axial turbine showcased superior power output and efficiency relative to the preceding models. Notably, the ducted-type axial turbine design achieved a C_p value of 0.98 at a TSR of 7.
• The Gate turbine highlighted the efficiency advantage of a 3-bladed design over a 4-bladed one, attributed to heightened pressure differences between the runner's pressure and suction sides. Moreover, a gate inclination angle of 14° emerged as the most efficient angle for power generation.
• It was observed that power output from the Gate turbine could be regulated by adjusting the gate inclination angle without significant efficiency loss.
• Despite the studied turbine designs, hydrokinetic turbines failed to meet target power and efficiency levels at the specified site.

The gate turbine showed a promising technology to harvest electricity from ultra-low head sites. The gate turbine is further investigated to find the optimum operating point and is an ongoing study. The future works include optimization of the RV design, cavitation analysis, manufacturing and testing the turbine at the real site and modifying the turbine to be site-independent such that the market scope is broadened. Improving the gate turbine design to incorporate the environmental friendliness nature of the turbine is currently under consideration. Although the Gate turbine is the only design that meets the target of this particular site, other turbine technologies can be installed at their appropriate sites with a lower target. This study aims to help turbine designers and manufacturers with an appropriate turbine selection.

## Data availability

Upon request, the corresponding author will provide the data supporting the study's conclusions.

## CRediT authorship contribution statement

**Mohamed Murshid Shamsuddeen:** Writing – review & editing, Writing – original draft, Visualization, Validation, Software, Methodology, Investigation, Formal analysis, Data curation, Conceptualization. **Mohammad Abu Shahzer:** Writing – review & editing, Software, Methodology, Investigation. **Min-Su Roh:** Validation, Investigation, Conceptualization. **Jin-Hyuk Kim:** Writing – review & editing, Supervision, Resources, Project administration, Funding acquisition.

## Declaration of competing interest

The authors declare that they have no known competing financial interests or personal relationships that could have appeared to influence the work reported in this paper.
